# Chemical composition and transcriptomic analysis revealed the dynamic changes of saponins during the growth of *Panax japonicus* var. *major*

**DOI:** 10.1093/aobpla/plaf034

**Published:** 2025-06-26

**Authors:** Yidan Peng, Xiujuan Peng, Jiayu Guo, Miaomiao Zhang, Yue Qin, Liang Peng, Yuqu Zhang, Ying Chen, Yonggang Yan, Gang Zhang, Juan Liu, Xinjie Yang

**Affiliations:** College of Pharmacy, Shaanxi University of Chinese Medicine, Xianyang 712046, China; Shaanxi Qinling Application Development and Engineering Center of Chinese Herbal Medicine, Xianyang 712046, China; Shaanxi Institute of International Trade & Commerce, Xianyang 712046, China; School of Pharmacy, Health Science Center, Xi’an Jiaotong University, Xi’an 710049, China; College of Pharmacy, Shaanxi University of Chinese Medicine, Xianyang 712046, China; Shaanxi Qinling Application Development and Engineering Center of Chinese Herbal Medicine, Xianyang 712046, China; College of Pharmacy, Shaanxi University of Chinese Medicine, Xianyang 712046, China; College of Pharmacy, Shaanxi University of Chinese Medicine, Xianyang 712046, China; Shaanxi Qinling Application Development and Engineering Center of Chinese Herbal Medicine, Xianyang 712046, China; College of Pharmacy, Shaanxi University of Chinese Medicine, Xianyang 712046, China; Shaanxi Qinling Application Development and Engineering Center of Chinese Herbal Medicine, Xianyang 712046, China; College of Pharmacy, Shaanxi University of Chinese Medicine, Xianyang 712046, China; Shaanxi Qinling Application Development and Engineering Center of Chinese Herbal Medicine, Xianyang 712046, China; College of Pharmacy, Shaanxi University of Chinese Medicine, Xianyang 712046, China; Shaanxi Qinling Application Development and Engineering Center of Chinese Herbal Medicine, Xianyang 712046, China; College of Pharmacy, Shaanxi University of Chinese Medicine, Xianyang 712046, China; Shaanxi Qinling Application Development and Engineering Center of Chinese Herbal Medicine, Xianyang 712046, China; College of Pharmacy, Shaanxi University of Chinese Medicine, Xianyang 712046, China; Shaanxi Qinling Application Development and Engineering Center of Chinese Herbal Medicine, Xianyang 712046, China; National Resource Center for Chinese Materia Medica, China Academy of Chinese Medical Sciences, State Key Laboratory for Quality Ensurance and Sustainable Use of Dao-di Herbs, Beijing 100700, China; College of Pharmacy, Shaanxi University of Chinese Medicine, Xianyang 712046, China; Shaanxi Qinling Application Development and Engineering Center of Chinese Herbal Medicine, Xianyang 712046, China; Phenome, Genome & Environment

**Keywords:** *Panax japonicus* var. *major*, transcriptome, saponins, biosynthesis, different growth years

## Abstract

*Panax japonicus* is a valuable medicinal plant whose rhizomes are rich in diverse ginsenosides. However, its perennial growth habit can significantly influence the quality and consistency of the herbal product. Despite its medicinal importance, the molecular regulatory mechanisms underlying saponin biosynthesis during different growth stages remain largely unknown. In this study, we conducted a comprehensive analysis of saponin content and transcriptomic profiles in *P. japonicus* rhizomes from plants aged 2–5 years. High-performance liquid chromatography revealed significant variations in saponin levels across different growth stages. Specifically, the concentrations of the major saponins, Ginsenoside Ro and Chikusetsusaponin IVa, decreased with increasing plant age, while the minor components, Zingibroside R1 and Calenduloside E, showed an upward trend. Transcriptome sequencing generated 78.53 Gb of clean reads and assembled 90 912 unigenes, of which 61 268 unigenes were successfully annotated. Comparative analysis indicated that *P. japonicus* shares the highest sequence homology with *Daucus carota* subsp. *sativa*. In addition, 37 enzymes involved in the triterpenoid saponin biosynthesis pathway were identified through differential gene expression analysis. Weighted Gene Co-expression Network Analysis further identified seven gene modules significantly associated with triterpenoid saponin content. Notably, genes encoding Cytochrome P450s and Uridine diphosphate-glycosyltransferases, which are key enzymes in saponin biosynthesis, were highlighted for further investigation. This study fills a critical knowledge gap in the genetic regulation of saponin biosynthesis in *P. japonicus* throughout its developmental stages and provides novel insights into the molecular mechanisms regulating ginsenoside accumulation. These findings offer a valuable foundation for future genetic improvement and quality control of *P. japonicus* as a traditional medicinal herb.

## Teaser

This study explores how the medicinal compounds in Panax japonicus var. major (a valuable herb) changed as the plant grew. Researchers analyzed rhizomes from 2- to 5-year-old P. japonicus and found that some key compounds decreased over time, while others increased. By studying the plant's genes, they identified which ones are involved in producing these compounds and how their activity changes with age. These findings help explain why the plant's medicinal properties vary with age and could guide better harvesting and cultivation methods.

## Introduction

*Panax japonicus* var. *major*, commonly known as ‘Da Ye San Qi’, is a perennial herb belonging to the genus *Panax* in the family Araliaceae. Its rhizome, also referred to as bead ginseng, is a highly valued traditional Chinese medicinal material (Chinese Pharmacopoeia Commission 2020; Ye et al. 2023). In China, *P. japonicus* is primarily distributed across eight provinces, including Gansu, Shaanxi, Henan, Yunnan, Guizhou, Sichuan, Tibet, and Hubei. It typically thrives in shaded, humus-rich soils at altitudes ranging from 1000 to 2200 m (Zhao 2015). In regions where *P. japonicus* is found, two other ginseng varieties, *Panax pseudoginseng* var. *bipinnatifidus* and *P. pseudoginseng* var. *Elegantior*, are also known to coexist (Peng et al. 2024). *Panax japonicus* exhibits a broad spectrum of pharmacological activities, including yin nourishment, cough relief, haemostasis, anti-fatigue, and sedative effects (Tang et al. 2020; Zhang et al. 2020). However, due to its lengthy growth cycle, the market supply of *P. japonicus* remains heavily reliant on wild harvesting. Overharvesting has led to a dramatic decline in wild populations, resulting in its designation as a nationally protected rare and endangered species (Wen and Zimmer 1996). The primary bioactive components in *Panax* species are triterpenoid saponins, which can be categorized into two major types based on their aglycone structures: dammarane-type saponins and oleanane-type saponins (Chen et al. 2020). Modern pharmacological studies have demonstrated the anti-tumour, antioxidant, anti-ulcer, haematopoietic, circulatory, and immunomodulatory properties of *P. japonicus* (Zhang et al. 2014; Shim et al. 2021). Notably, individual saponins exhibit distinct pharmacological activities. For example, Chikusetsusaponin IVa exhibits strong inhibitory effects on adipocyte differentiation (Yin et al. 2018), while Ginsenoside Rb1 has demonstrated anti-diabetic activity (Zhou et al. 2019) and protective effects against OGD/R-induced astrocyte injury (Liu et al. 2023).

The biosynthesis of saponins in *P. japonicus* primarily follows the mevalonate (MVA) pathway (Li et al. 2023). This pathway begins with the conversion of acetyl-CoA to mevalonate, catalysed by acetyl-CoA acyltransferase (AACT) and 3-hydroxy-3-methylglutaryl-CoA reductase (HMGR). Subsequently, isopentenyl pyrophosphate and dimethylallyl pyrophosphate are synthesized and further condensed by farnesyl pyrophosphate synthase to form farnesyl pyrophosphate (FPP). FPP is then converted to 2,3-oxidosqualene through the catalytic actions of squalene synthase (SS) and squalene epoxidase (Tang et al. 2019; An et al. 2022). At this point, the biosynthetic pathway diverges: 2,3-oxidosqualene is cyclized into dammarenediol via dammarenediol synthase in the dammarane-type saponin pathway and into β-amyrin via β-amyrin synthase (β-AS) in the oleanane-type saponin pathway (Jo et al. 2017). However, the exact regulatory mechanisms and enzymatic steps governing these downstream pathways remain to be fully characterized.

Transcriptomics enables the investigation of gene expression at the transcriptional level, playing a crucial role in identifying key genes involved in metabolite biosynthetic pathways. Previous studies on *P. japonicus* have primarily focused on transcriptome differences across various tissues. Notably, a number of uridine diphosphate-glycosyltransferase (UGT) genes involved in triterpenoid saponin biosynthesis have been found to be preferentially expressed in rhizome nodes. Zhang et al. (2020) reported that many UGT genes in the triterpene saponin biosynthesis pathway of *P. japonicus* are preferentially expressed in the rhizome nodes. Additionally, transcriptomic analysis has been employed to identify candidate genes potentially associated with the development and expansion of rhizome nodes in *P. japonicus* (Tang et al. 2020). Yang et al. (2024a) employed chemical and transcriptomic analyses to identify saponin biosynthesis genes in the periderm, cortex, and stele of *P. japonicus* rhizomes. Among these, a candidate UGT gene was proposed to catalyse the glycosylation of Chikusetsusaponin IVa in *P. japonicus*.

The pharmacological activities of medicinal plants are primarily attributed to their secondary metabolites, whose accumulation is influenced by various factors, including growth cycle, temperature, and other environmental conditions. In *Panax* species such as *Panax ginseng* (Liu et al. 2017a), *Panax notoginseng* (Yan et al. 2024), and *P. japonicus* C. A. Mey (Huang et al. 2023), the content of triterpenoid saponins varies significantly with plant age. These variations lead to substantial differences in quality evaluation, clinical efficacy, and commercial application across growth stages. However, few studies have systematically explored the molecular mechanisms underlying the dynamic changes in metabolite accumulation during the growth and development of *P. japonicus*. Transcriptomic and metabolomic analyses provide powerful tools to address biological issues related to gene expression and metabolite accumulation, respectively (Yang et al. 2024b). High-performance liquid chromatography (HPLC) enables precise quantification of active components, thereby facilitating the monitoring of metabolite dynamics throughout developmental stages. When combined with transcriptomic data, this approach allows for the identification of key gene expression changes associated with metabolite biosynthesis. Therefore, it is essential to transcriptomically investigate the underground tissues of *P. japonicus* at different growth stages. Such research will provide a scientific foundation for the conservation, introduction, domestication, and artificial cultivation of this valuable medicinal resource.

To investigate the molecular mechanisms underlying saponin biosynthesis in *P. japonicus*, we employed an integrated approach combining transcriptomic analysis and HPLC. This study comprehensively examined the dynamic changes in ginsenoside content and gene expression patterns in *P. japonicus* rhizomes across developmental stages from 2 to 5 years of age. The results revealed significant variation in ginsenoside content throughout development and identified a preliminary association between terpenoid biosynthesis-related genes and ginsenoside accumulation in rhizomes. These findings provide a scientific foundation for the rational exploitation, resource management, and optimization of harvest timing for *P. japonicus*.

## Materials and methods

### Plant materials

The rhizomes of *P. japonicus* (DY) were collected from the cultivation base of *P. japonicus* (via rhizome propagation) in the Qinling Red River Valley Forest Park, Baoji, Shaanxi, China, at coordinates (34° N 19′ 0″, 108° E 42′ 50″; altitude: 2300 m). Samples aged 2–5 years were collected, with four groups for this study, each consisting of three biological replicates. The 2-, 3-, 4- and 5-year-old *P. japonicus* samples were designated as DY2-1, DY2-2, DY2-3, DY3-1, DY3-2, DY3-3, DY4-1, DY4-2, DY4-3, DY5-1, DY5-2, and DY5-3. All samples were washed with purified water, cut into small pieces, rapidly frozen in liquid nitrogen, and stored in an ultra-low temperature refrigerator at −80°C for RNA extraction. The remaining samples were used for saponin content determination by HPLC. The identification of the *P. japonicus* samples was confirmed by Professor Yonggang Yan from Shaanxi University of Chinese Medicine, and a voucher specimen was deposited in the Herbarium of Shaanxi University of Chinese Medicine.

### Determination of saponin contents by the high-performance liquid chromatography method

The rhizomes of *P. japonicus* at four different ages were washed, shade-dried, ground, and sieved separately through an 80-mesh sieve. The measurement of saponin-like compounds was optimized following the method introduced by Zhang et al. (2020). Specifically, 0.15 g of dried powder was accurately weighed and extracted with 50 ml of methanol. The suspension was subjected to ultrasonic treatment for 45 min at 40 Hz. Afterward, the samples were filtered through a 0.22 μm filter membrane (Green Mall, China). Subsequently, 10 μl of the filtrate was injected into an LC-2030C 3D Plus HPLC system equipped with a PDA detector and an automatic sampler (Shimadzu, Japan). Separation was achieved using HPLC on an Agilent 5 TC-C18 column (250 mm × 4.6 mm, 5 μm) at 30°C with detection at 203 nm. A binary solvent gradient was employed, consisting of Solvent A (acetyl) and Solvent B (0.2% water phosphate). The gradient elution procedure was performed with A and B as follows: 20%–28% A with 80%–72% B from 0 to 13 min, 28%–44% A with 72%–56% B from 13 to 30 min, 44%–48% A with 56%–52% B from 30 to 50 min, 48%–51% A with 52%–49% B from 50 to 60 min, and 51%–60% A with 49%–40% B from 60 to 65 min.

Qualitative analysis of *P. japonicus* was completed by comparing the retention times with the standard substances Ginsenoside Re, Rb1, Ro, Rd, Chikusetsusaponin IVa, Zingibroside R1, and Calenduloside E (Yuanye Biotechnology, Shanghai, China). To comply with HPLC analysis standards, the purity of all standards was found to exceed 98%. Saponin content was determined according to the standard curves: Ginsenoside Re: *y* = 460 611.3404*x* + 8609.5854, *R*^2^ = 0.9999; Ginsenoside Rb1: *y* = 194 737.7379*x* + 192.7561, *R*^2^ = 0.9998; Ginsenoside Ro: *y* = 313 324.4116*x* + 289 999.4146, *R*^2^ = 0.9990; Ginsenoside Rd: *y* = 275 798.2444*x* − 539.0244, *R*^2^ = 1.0000; Chikusetsusaponin IVa: *y* = 422 828.1233*x* + 20 148.7561, *R*^2^ = 0.9998; Zingibroside R1: *y* = 374 634.5930*x* − 6742.1951, *R*^2^ = 0.9993; Calenduloside E: *y* = 436 537.1987*x* − 570.7317, *R*^2^ = 0.9995. GraphPad Prism 8.0 software was used for the significant analysis, and Adobe Photophoto CC software (Adobe Photoshop CC 2019) was employed for image organization.

### Transcriptome sequencing analysis

Total RNA was extracted using the RNAprep Pure Plant Kit (DP441) (TIANGEN, Beijing, China). The quality of the RNA was assessed using 1% agarose gels and a NanoPhotometer® spectrophotometer (Implen, CA, USA). Qubit3.0 fluorescence (Invitrogen, CA, USA) and Agilent 5400 Bioanalyzer (Agilent Technologies, CA, USA) were adopted for the identification and quantification of the total RNA. Qualified RNA from each sample was converted into cDNA libraries using the NEBNext® Ultra™ II RNA Library Prep Kit for Illumina® (NEB, MA, USA). The cDNA libraries were diluted to 1.5 ng/µl and analysed on the Agilent 5400 Bioanalyzer. The effective concentration of the library was accurately quantified using the q-PCR method. RNA sequencing was conducted on an Illumina NovaSeq 6000 (Illumina, CA, USA), and the quality control of raw reads was performed using Fastp (version 0.23.2). Trinity (https://github.com/trinityrnaseq/trinityrnaseq) uses paired-end splicing on single transcriptome library sequences to cluster, integrate, and extend contigs, resulting in de-redundant transcripts (unigene). The unigenes were compared with Non-Redundant (NR), Kyoto Encyclopedia of Genes and Genomes (KEGG), SwissProt, EuKaryotic Orthologous Groups (KOG), Gene Ontology (GO), Tremble, and Pfam databases using DIAMOND software (diamond v 0.9.24.125; Diamond Software Systems Ltd, London, UK). The HMMER software (HMMER 3.2 package) was employed to obtain annotation information of seven transcript databases. The expression levels of transcripts were standardised for fragments per thousand bases to obtain fragments per kilobase of exon model per million mapped fragments (FPKM).

For samples with biological replicates, gene expression profile analysis between sample groups was performed using DESeq2 (v1.38.3). All samples were analysed in pairwise comparisons (DY2_vs_DY3, DY2_vs_DY4, DY2_vs_DY5, DY3_vs_DY4, DY3_vs_DY5, and DY4_vs_DY5) to obtain differentially expressed genes (DEGs). DEGs were identified with a false discovery rate of <0.05 and |log_2_-fold change| of ≥1. KOG annotation was performed by aligning gene sequences against the KOG database (https://ftp.ncbi.nih.gov/pub/COG/KOG/) using Diamond-v2.0.9. The alignment parameters were set as follows: -p 8-f 6-e 1e-5-max-hsps 1-sensitive-k 1-b 8-c 1. The MISA v1.0 software was utilized for the analysis of the simple sequence repeat (SSR) loci. The minimum number of repeat units for mononucleotide to hexanucleotide repeats was set at 10, 6, 5, 5, 5, and 5, respectively. The maximum interruption between two SSRs was set at 100, with other parameters set to default. Transcription factors (TFs) in unigenes were identified using iTAK (v1.6) software. WGCNA was performed using the R software (v4.2.2) and the WGCNA package (v1.71) on the Cloud platform analysis tools (https://cloud.metware.cn).

## Results

### Quantitative analysis of ginsenosides in rhizomes of *Panax japonicus*

**Figure 1.**
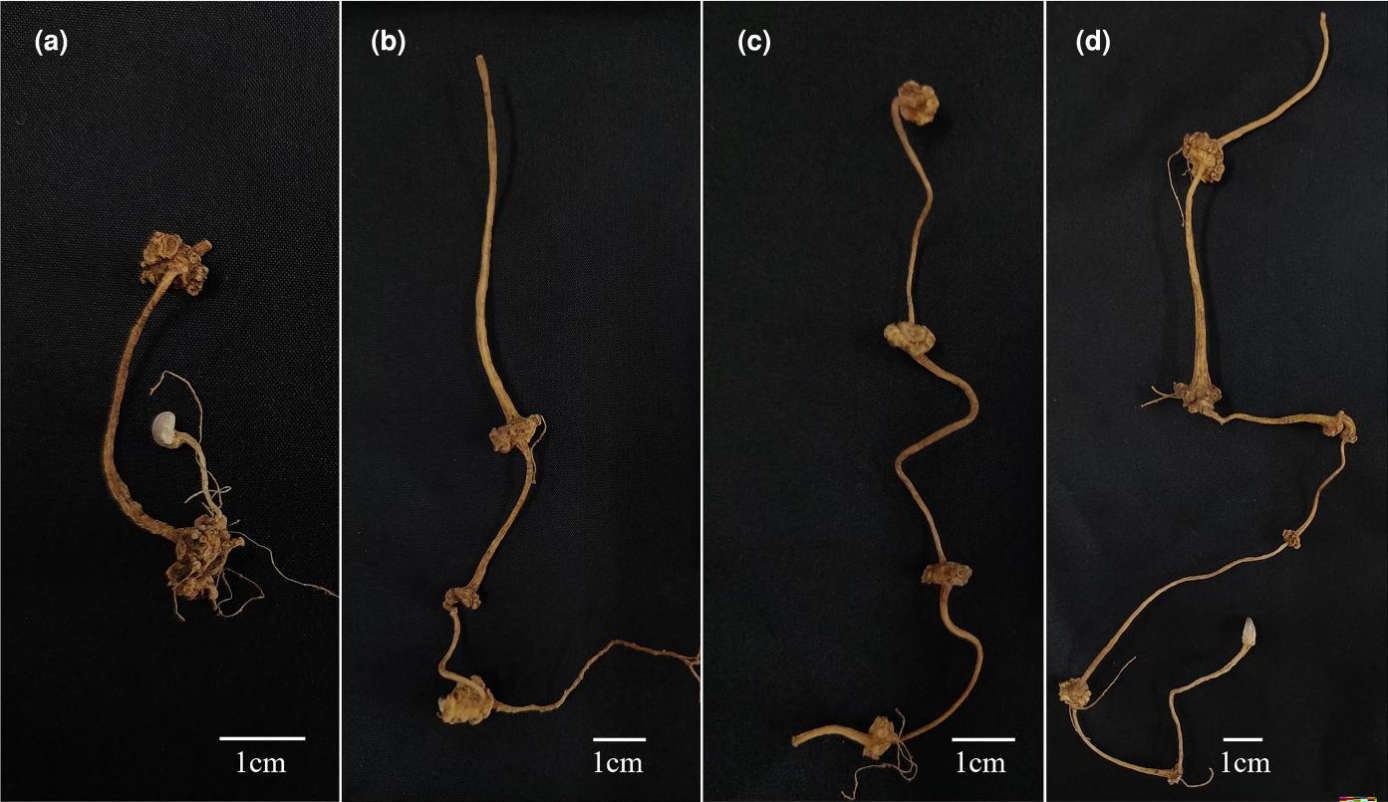
Phenotypic characteristics in *P. japonicus* underground tissues. (a-d) represent samples of 2, 3, 4, and 5 years old, respectively.

**Figure 2.**
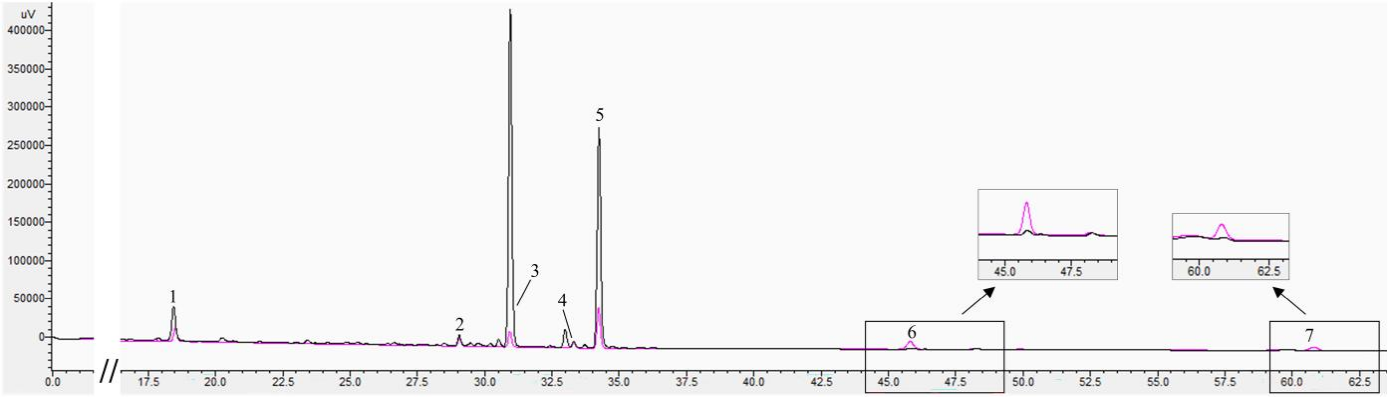
HPLC profiles of ginsenosides in the rhizome of *P. japonicus*. Determination of seven ginsenosides: Ginsenoside Re (1), Ginsenoside Rb1 (2), Ginsenoside Ro (3), Ginsenoside Rd (4), Chikusetsusaponin IVa (5), Zingibroside R1 (6) and Calenduloside E (7).

**Figure 3.**
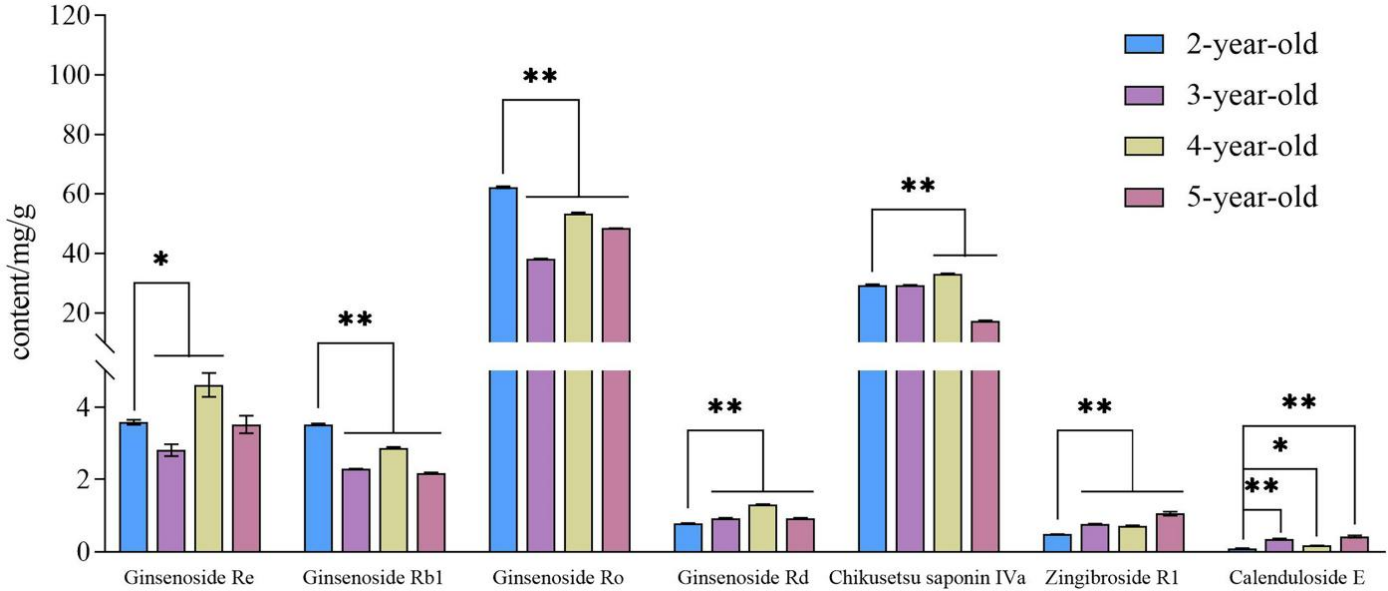
Contents of seven ginsenosides from different ages of *P. japonicus* (mg/g). *n* = 3. *P < .05, **P < .01.

Four groups of *P. japonicus* rhizome samples, representing 2- to 5-year-old plants, were collected for analysis (Fig. 1). The external morphology of the rhizomes is characterized by slender internodes and nodal swellings, which give them a rosary-like appearance. The HPLC chromatogram used to determine ginsenoside content in the rhizomes is presented in Fig. 2, and the quantified contents of seven ginsenosides across different growth stages are shown in Fig. 3. The results clearly indicate a decreasing trend in the concentrations of Ginsenoside Rb1, Ginsenoside Ro, and Chikusetsusaponin IVa with increasing plant age, while the levels of Zingibroside R1 and Calenduloside E show a marked increase. In contrast, the contents of Ginsenoside Re and Ginsenoside Rd remain relatively stable throughout the developmental stages. Notably, Ginsenoside Ro and Chikusetsusaponin IVa are the most abundant saponins in the rhizomes of *P. japonicus*, accounting for 51.11%–65.49% and 23.54%–39.32% of the total quantified saponins, respectively.

### Transcriptome sequencing and data assembling

Transcriptome sequencing of *P. japonicus* produced a total of 78.53 Gb of clean data. The Q20 scores for the clean reads from all experimental samples exceeded 96.88%, while the Q30 scores were >91.64% (Table 1). Q30 values reflect the base calling accuracy, with higher values indicating lower error rates and greater confidence in sequence quality. These results demonstrate that the sequencing yielded high-quality clean reads, thereby providing reliable data for downstream gene analysis. Following *de novo* assembly, a total of 146 117 unigenes were obtained.

**Table 1.** Summary statistics of the RNA-seq results.

Sample	Raw reads	Clean reads	Clean read rate (%)	Clean base (G)	Error rate (%)	Q20 (%)	Q30 (%)	GC content (%)
DY2-1	50207384	42106992	84	6.32	0.02	98.22	94.46	42.91
DY2-2	57424164	55776924	97	8.37	0.03	97.22	91.90	42.99
DY2-3	47448926	40295188	85	6.04	0.03	98.02	94.07	43.44
DY3-1	52286912	44314084	85	6.65	0.02	98.13	94.25	43.25
DY3-2	47810542	39729910	83	5.96	0.03	97.86	93.64	43.46
DY3-3	46789568	38944950	83	5.84	0.02	98.17	94.44	43.37
DY4-1	46386092	42041582	91	6.31	0.03	98.07	94.20	43.36
DY4-2	44396822	41171548	93	6.18	0.02	98.12	94.34	43.74
DY4-3	47335704	43323118	92	6.50	0.02	98.22	94.59	43.46
DY5-1	48999020	46367312	95	6.96	0.03	97.13	91.76	43.24
DY5-2	46062696	41829472	91	6.27	0.03	98.02	94.05	43.88
DY5-3	48657958	47543962	98	7.13	0.03	96.88	91.64	43.82

### Functional annotation and expression analysis of unigenes

Among the 146 117 unigenes obtained, 61 871 (42.34%) were annotated in KEGG, including 82 743 (56.63%) in Nr, 58 488 (40.03%) in SwissProt, 84 332 (57.72%) in TrEMBL, 51 664 (35.36%) in KOG, 74 344 (50.88%) in GO, and 50 833 (34.79%) in Pfam. In total, 89 428 unigenes (61.20%) were successfully annotated in at least one database (Table 2).

**Table 2.** Unigenes annotation of *P. japonicus*.

Database	Number of genes	%
KEGG	61 871	42.34
Nr	82 743	56.63
SwissProt	58 488	40.03
TrEMBL	84 332	57.72
KOG	51 664	35.36
GO	74 344	50.88
Pfam	50 833	34.79
Annotated in at least one database	89 428	61.20
Total unigenes	146 117	100.00

**Figure 4.**
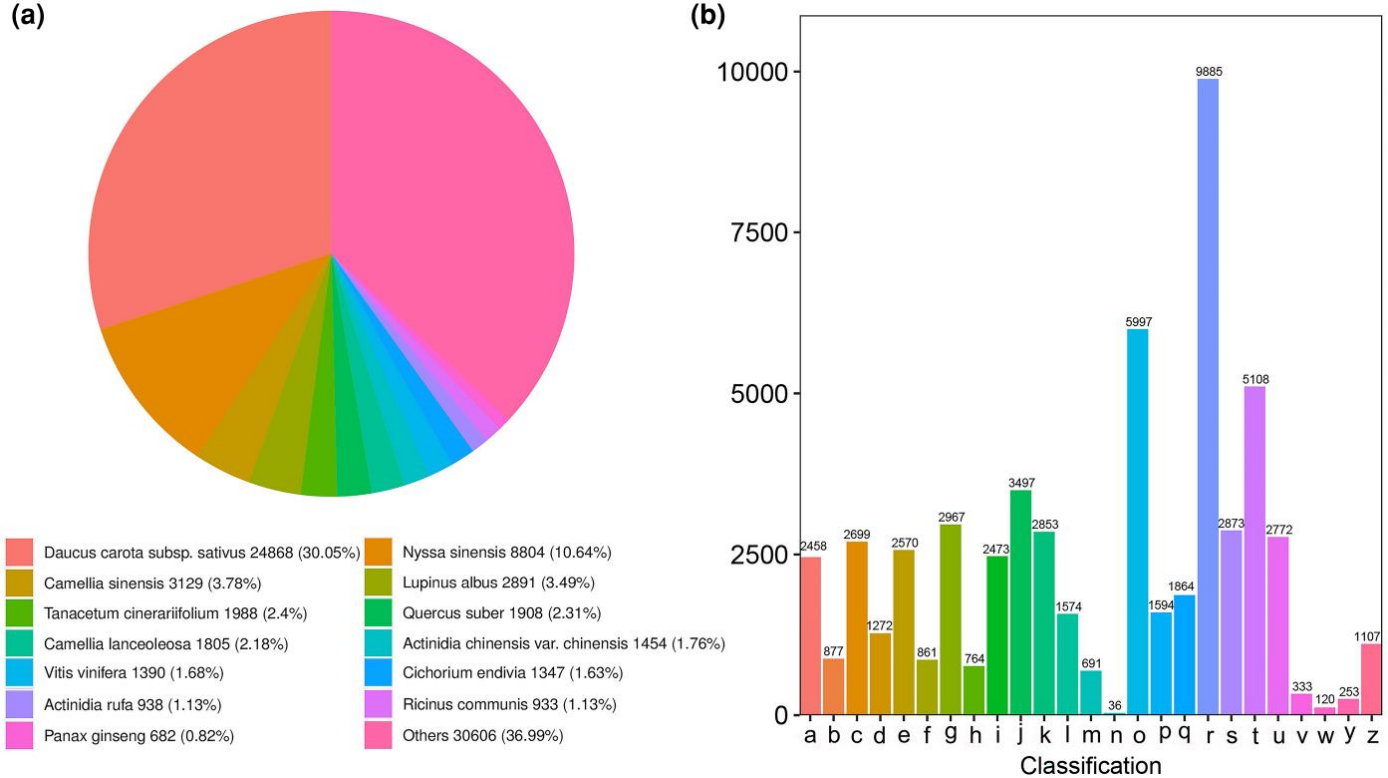
Functional annotation of unigenes. (a) Species distribution annotated in the NR database. (b) KOG annotation classification statistical map of genes. a: RNA processing and modification; b: chromatin structure and dynamics; c: energy production and conversion; d: cell cycle control, cell division, and chromosome partitioning; e: amino acid transport and metabolism; f: nucleotide transport and metabolism; g: carbohydrate transport and metabolism; h: coenzyme transport and metabolism; i: lipid transport and metabolism; j: translation, ribosomal structure, and biogenesis; k: transcription; l: replication, recombination, and repair; m: cell wall/membrane/envelope biogenesis; n: cell motility; o: post-translational modification, protein turnover, chaperones; p: inorganic ion transport and metabolism; q: secondary metabolites biosynthesis, transport, and catabolism; r: general function prediction only; s: function unknown; t: signal transduction mechanisms; u: intracellular trafficking, secretion, and vesicular transport; v: defence mechanisms; w: extracellular structures; y: nuclear structure; z: cytoskeleton.

Unigene sequences were compared against the NR database to identify homologous sequences in closely related species (Fig. 4a). The highest sequence similarity was observed with *Daucus carota* subsp*. sativa*, accounting for 24 868 unigenes (30.05%), followed by *Nyssa sinensis* (8804; 10.64%), *Camellia sinensis* (3129; 3.78%), and *Lupinus albus* (2891; 3.49%). These annotations provide valuable references for the functional study of genes in *P. japonicus*.

The KOG database classifies orthologous genes based on evolutionary and functional relationships. Unigenes belonging to the same orthologous group are presumed to share functional similarity. In this study, 51 664 unigenes were assigned to 25 KOG categories (Fig. 4b). Among them, the largest group was ‘general function prediction only’ (9885 unigenes, 19.13%), followed by ‘posttranslational modification, protein turnover’ (5997; 11.61%) and ‘signal transduction mechanisms’ (5108; 9.89%).

### Quantification of gene expression

**Figure 5.**
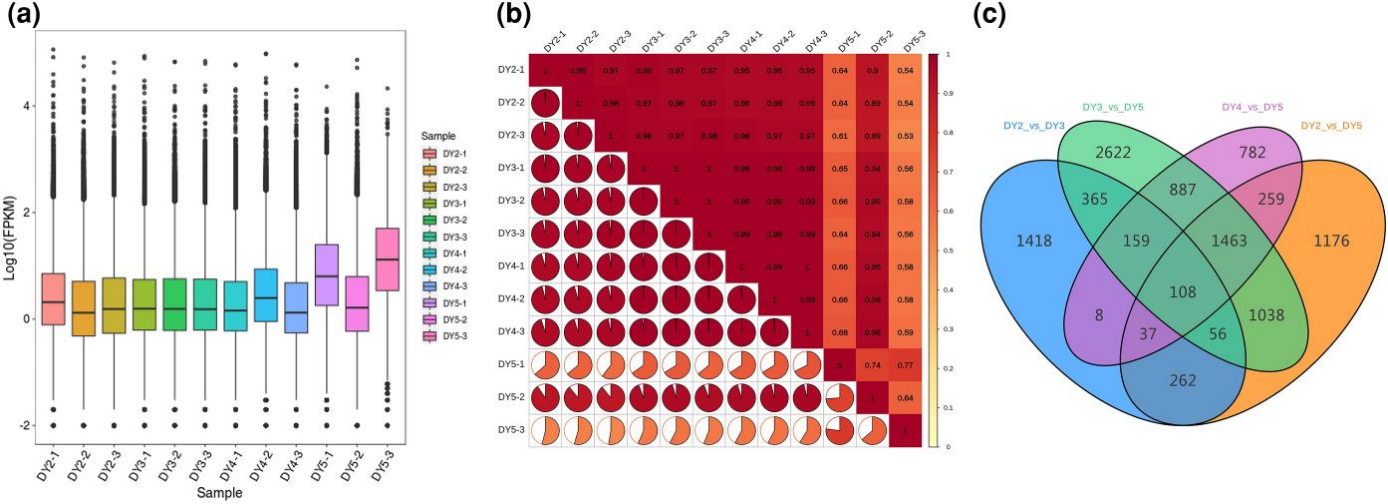
Preliminary analysis of transcriptome data. (a) Box plot of unigenes expressed in the ‘four’ groups of the *P. japonicus* samples, presenting the distributions of expression levels. (b) The square values of Pearson’s correlation coefficients (R2). (c) Venn diagram of DEG.

PKM is defined as the number of reads per kilobase of transcript length per million total reads (Cole et al. 2010). A box plot of gene expression levels (Fig. 5a) across different FPKM intervals demonstrated variability among the four *P. japonicus* samples, indicating differences in transcript abundance. Pearson correlation analysis revealed strong overall similarity among the samples, as visualized in the lower triangular heat map (Fig. 5b). In this heat map, deeper red colouration corresponds to higher correlation values. Notably, the 5-year-old samples showed a distinct separation from the others, suggesting increased transcriptional activity associated with prolonged growth. Additionally, a Venn diagram (Fig. 5c) was constructed to display the number of overlapping DEGs across various pairwise comparison groups, providing further insight into gene expression changes at different developmental stages.

### Differential expression analysis of *Panax japonicus* samples

**Figure 6.**
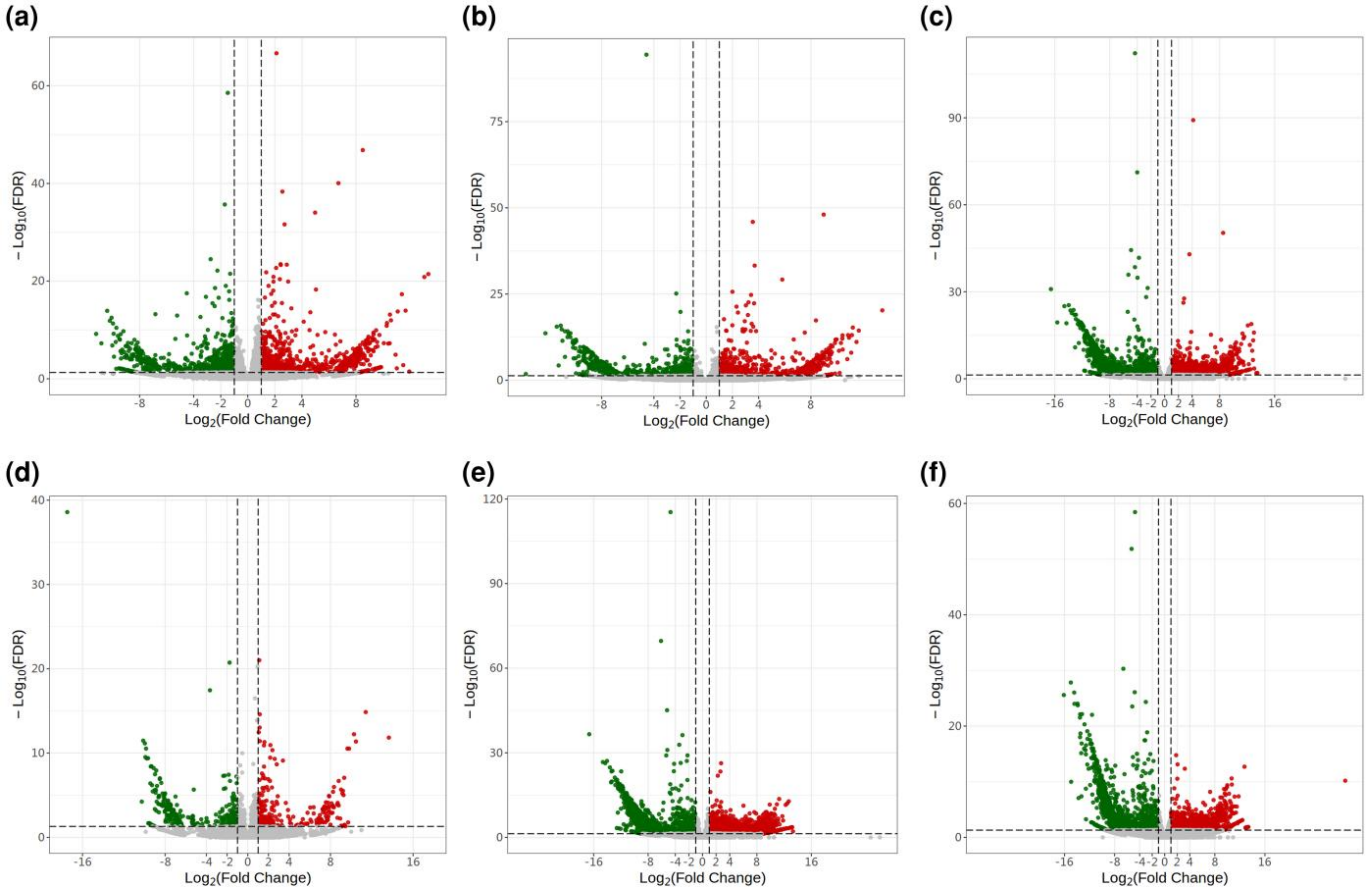
Volcano plot of DEGs. (a) DY_2-vs-DY_3; (b) DY 2-vs-DY_4; (c) DY_2-vs-DY_5; (d) DY_3-vs-DY_4; (e) DY_3-vs-DY_5; (f) DY_4-vs-DY_5.

A total of 11 358 genes were expressed in at least one sample with FPKM > 0 (Table S1). Differential expression analysis revealed 2413 DEGs between the DY2_vs_DY3 group, including 1158 upregulated and 1255 downregulated, as identified by RNA sequencing (Fig. 6). In the DY2_vs_DY4 group, 1451 DEGs were detected, with 717 upregulated and 734 downregulated. Notably, the DY2_vs_DY5 comparison yielded 4399 DEGs, comprising 1919 upregulated and 2480 downregulated. In contrast, only 616 DEGs were found in the DY3_vs_DY4 group, including 306 upregulated and 310 downregulated. The largest number of DEGs was observed in the DY3_vs_DY5 group, with 3403 upregulated and 3295 downregulated. For the DY4_vs_DY5 group, a total of 3703 DEGs were identified, with 1864 upregulated and 1839 downregulated.

### Overrepresentation analyses of differentially expressed genes in different growth years of *Panax japonicus*

**Figure 7.**
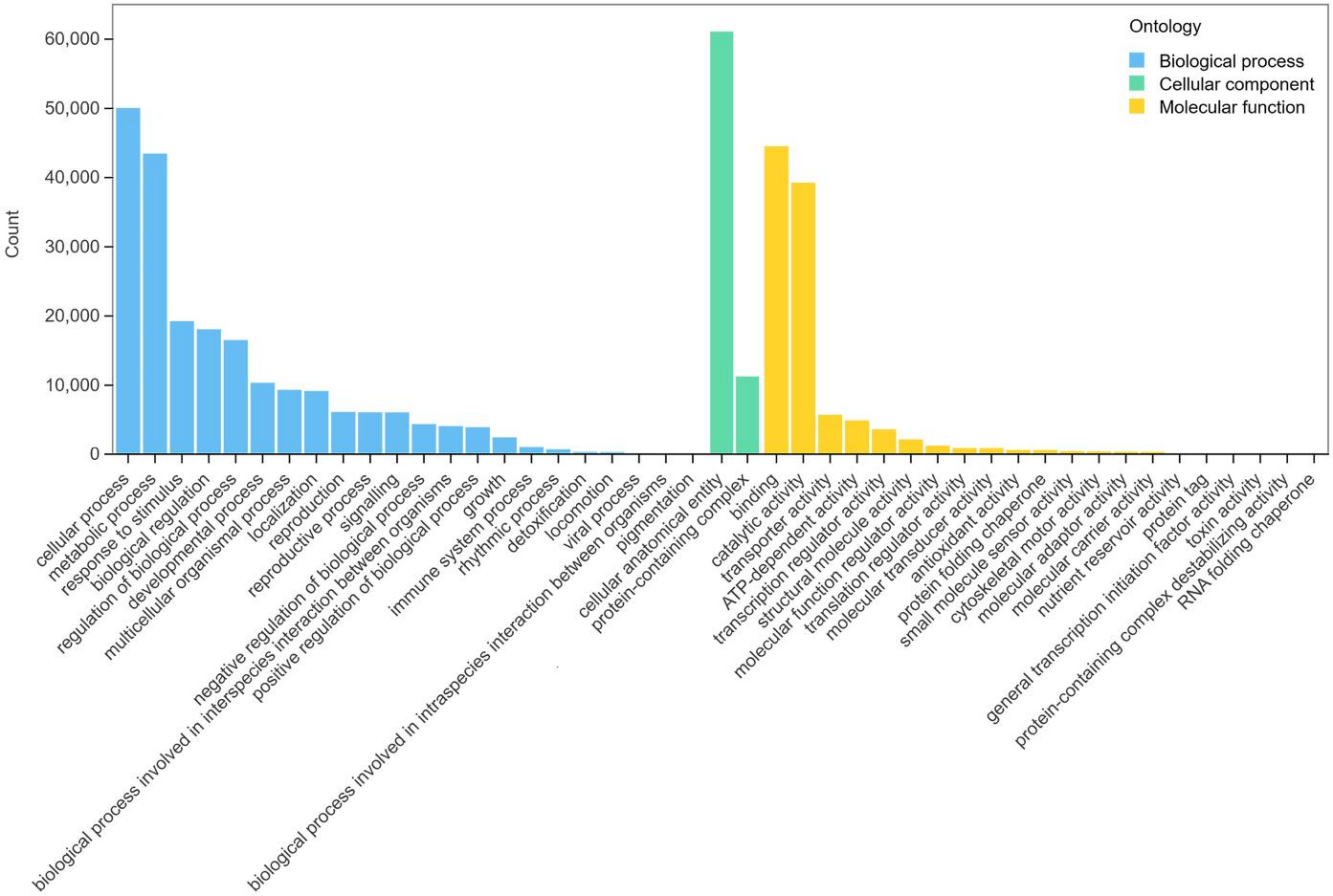
Gene ontology classification of the assembled unigenes from the *P. japonicus* transcriptome.

The GO database provides a standardized framework for biological annotation. A total of 74 344 unigenes from *P. japonicus* were annotated using the GO database and classified into three major categories: cellular component (72 358 annotations, 18.52%), molecular function (106 198 annotations, 27.19%), and biological process (212 042 annotations, 54.29%). Within the cellular components, the ‘cellular anatomical entity’ group represented the highest proportion, comprising 61 089 entries. The unigenes associated with molecular function were divided into 21 subcategories, among which ‘binding’ (44 533 entries) and ‘catalytic activity’ (39 262 entries) were the most abundant classes. In the biological process category, unigenes were assigned to 22 groups, with ‘cellular process’ (50 057 entries) and ‘metabolic process’ (43 471 entries) being the largest subcategories (Fig. 7; Table S2).

**Figure 8.**
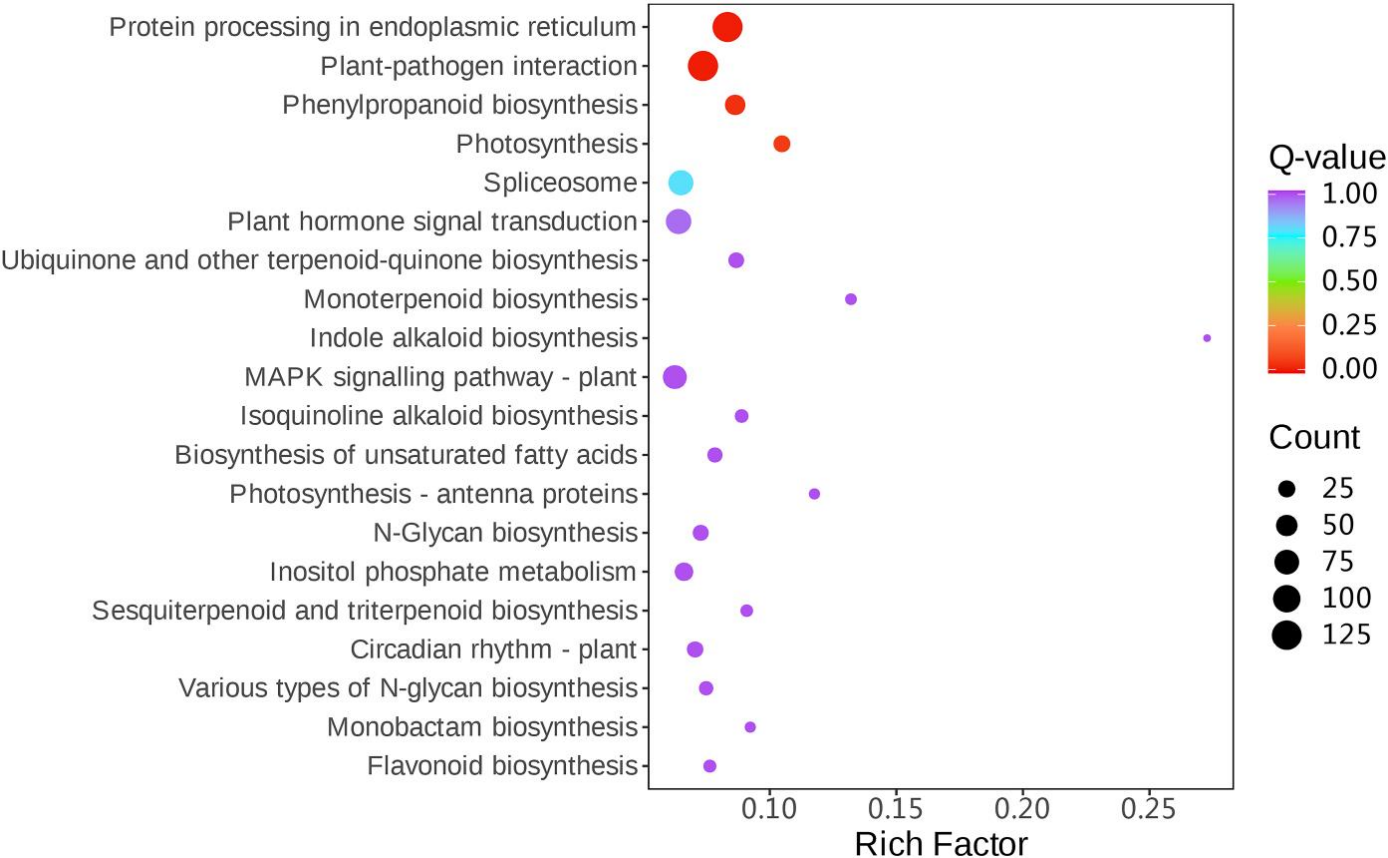
KEGG enrichment dot plot of DEGs between 2- and 5-year-old *P. japonicus*.

The KEGG database encompasses a broad spectrum of metabolic pathways and biochemical reactions, making it a crucial resource for understanding metabolic networks and regulatory mechanisms that govern various physiological and biochemical processes in plants. Through KEGG annotation analysis, 61 871 unigenes from *P. japonicus* were mapped to 142 pathways. These pathways were primarily classified into five categories: metabolism, genetic information processing, cellular processes, organismal systems, and environmental information processing (Table S3). The majority of unigenes were enriched in metabolic pathways. Furthermore, a total of 81 DEGs were found to be significantly enriched in five metabolic pathways associated with terpene biosynthesis across various comparison groups (Table 3). Notably, the highest number of upregulated DEGs was observed in the DY2_vs_DY5 comparison. The dot plot (Fig. 8) illustrates that the most actively enriched pathways in this group include protein processing in the endoplasmic reticulum, plant-pathogen interaction, phenylpropanoid biosynthesis, photosynthesis, spliceosome, plant hormone signal transduction, ubiquinone and other terpenoid-quinone biosynthesis, and monoterpenoid biosynthesis. These findings suggest that genes related to the terpenoid biosynthetic pathway become increasingly active with plant age.

**Table 3.** Number of genetic enzymes involved in the upregulation of terpene biosynthesis in four rhizome samples of different ages.

Metabolic pathway	Pathway number	DY2 -vs-DY3	DY2 -vs-DY4	DY2 -vs-DY5	DY3 -vs-DY4	DY3 -vs-DY5	DY4 -vs-DY5
Terpenoid backbone biosynthesis	ko00900	3	2	8	0	0	4
Monoterpenoid biosynthesis	ko00902	2	1	3	0	0	2
Diterpenoid biosynthesis	ko00904	1	0	1	0	3	3
Sesquiterpenoid and triterpenoid biosynthesis	ko00909	3	0	3	1	3	4
Ubiquinone and other terpenoid-quinone biosynthesis	ko00130	3	2	9	2	11	7
Total		12	5	24	3	17	20

### Pathway for the synthesis of *Panax japonicus* saponins

**Figure 9.**
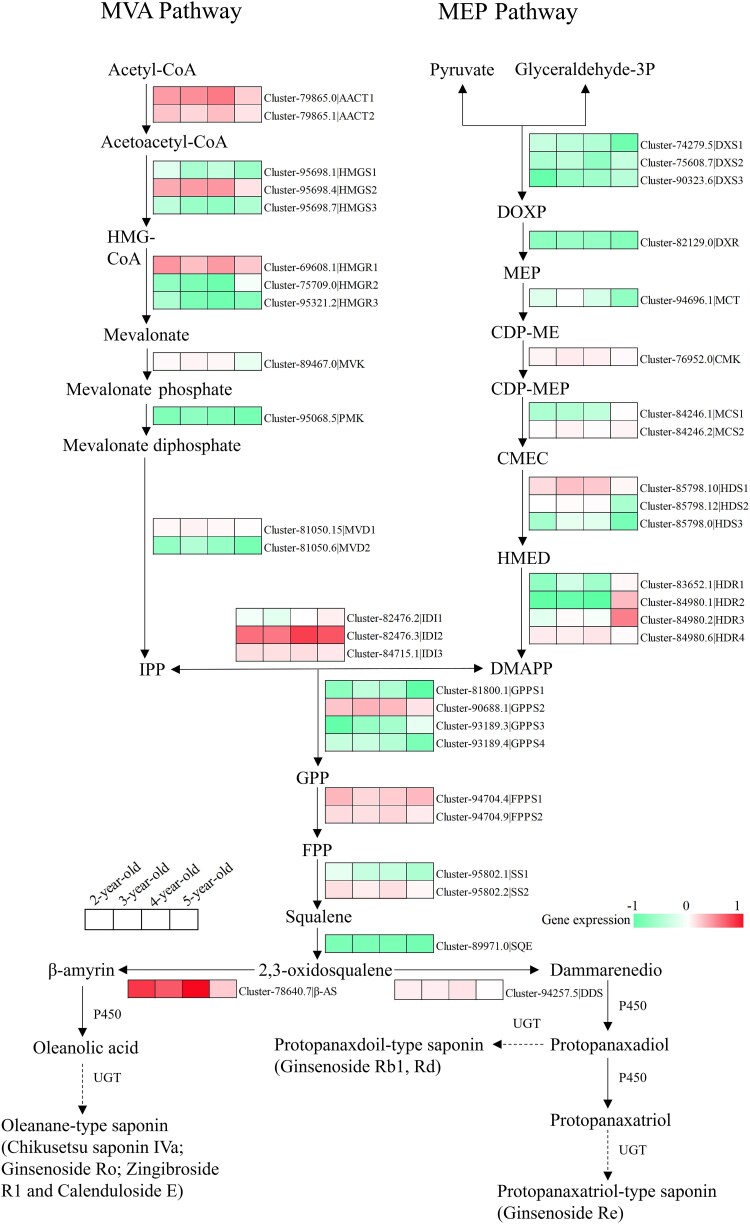
*Panax japonicus* saponins biosynthesis pathway and gene expression level with different growth years. AACT, acetyl-CoA acetyltransferase; HMGS, hydroxymethylglutaryl-CoA synthase; HMGR, hydroxymethylglutaryl-CoA reductase; MVK, mevalonate kinase; PMK, phosphomevalonate kinase; MVD, mevalonate-5-diphosphate decarboxylase; DXS, 1-deoxy-D-xylulose-5-phosphate synthase; DXR, 1-deoxy-D-xylulose-5-phosphate reductoisomerase; MCT, 4-cytidine diphosphate-methyl-D-erythritol synthase; CMK, 4-diphosphocytidyl-2-*C*-methyl-D-erythritol kinase; MCS, 2-*C*-methyl-D-erythritol-2,4-cyclodiphosphate synthase; HDS, (E)-4-hydroxy-3-methylbut-2-enyl-diphosphate synthase; HDR, 4-hydroxy-3-methylbut-2-enyl-diphosphate reductase; IDI, isopentenyl-diphosphate delta-isomerase; SS, squalene synthase; SQE, squalene epoxidases; β-AS, β-amyrin synthases; DDS, dammarenediol-II synthase.

To further investigate the gene regulatory network involved in the biosynthesis of triterpenoid saponins in *P. japonicus*, we analysed the expression patterns of genes associated with this pathway. A total of 37 genes related to terpenoid backbone biosynthesis were identified and categorized into three key metabolic pathways: the MVA pathway, the MEP pathway, and the 2,3-oxidosqualene pathways (Fig. 9). *AACT1* (Cluster-79865.0), *AACT2* (Cluster-79865.1), *HMGS2* (Cluster-95698.4), *HMGR1* (Cluster-69608.1), *HDS1* (Cluster-85798.10), *IDI2* (Cluster-82476.3), *IDI3* (Cluster-84715.1), *GPPS2* (Cluster-90688.1), *FPPS1* (Cluster-94704.4), *FPPS2* (Cluster-94704.9), and *β-AS* (Cluster-78640.7) showed higher expression levels, with their peak expressions observed predominantly in 2- or 4-year-old *P. japonicus* samples.

**Figure 10.**
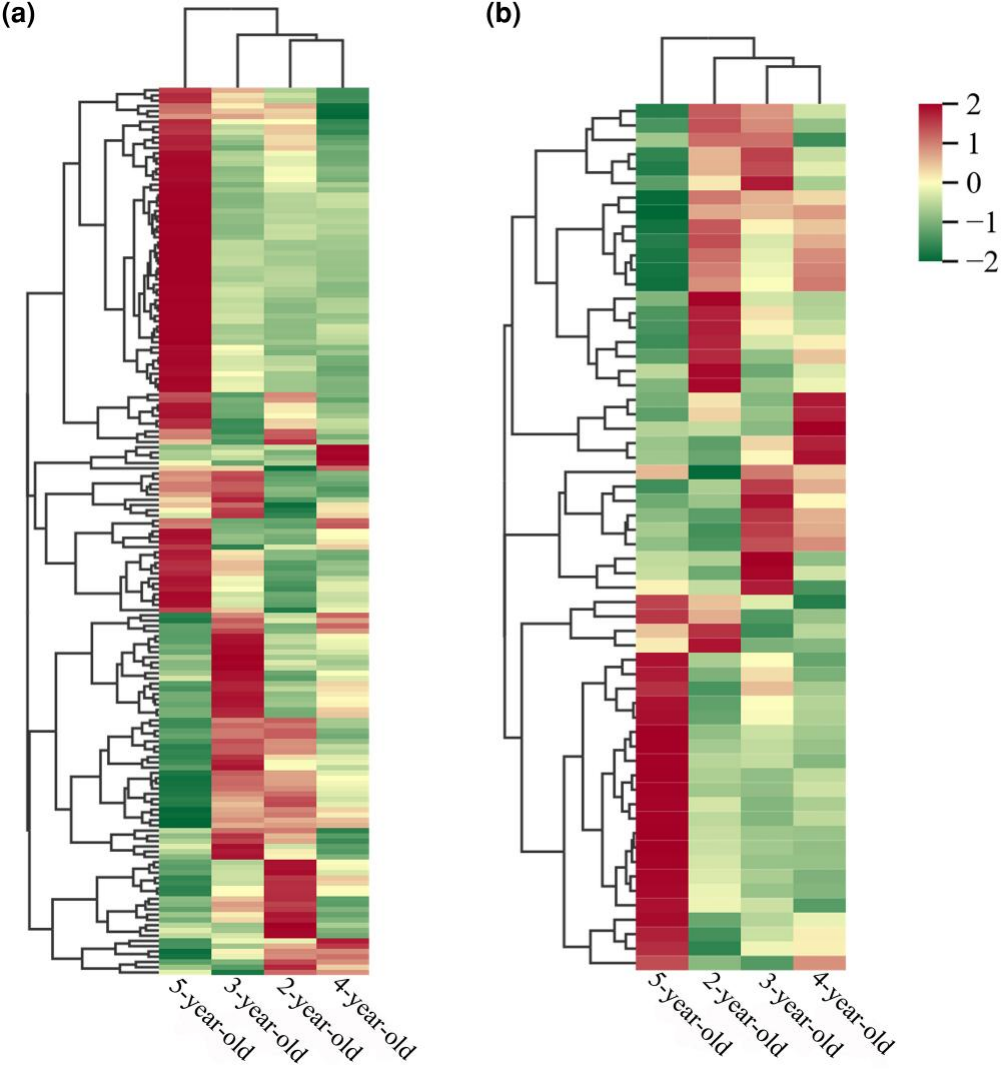
Expression of CYP and UGT genes in the biosynthesis of saponins in 2- to 5-year-old *P. japonicus*. (a) The expression patterns heat map of CYP genes. (b) The expression patterns heat map of UGT genes (see Table S4 for a list of codes and names).

Cytochrome P450s (CYPs) and UGTs are key downstream genes involved in regulation of triterpenoid saponin biosynthesis (Han et al. 2011, 2012). The differential expression of these genes may contribute to the age-related variation in saponin content observed in *P. japonicus*. In this study, CYP and UGT transcripts with FPKM values >0.3 were identified from the transcriptome data. A total of 169 CYP genes were identified, with peak expression observed in 80 (47.34%) from 5-year-old, 45 (26.63%) from 3-year-old, 32 (18.93%) from 2-year-old, and 12 (7.1%) from 4-year-old rhizome samples (Fig. 10a; Table S4). Similarly, 60 UGT genes were identified, with maximum expression found in 24 (40%) from 5-year-old, 17 (28.33%) from 2-year-old, 12 (20%) from 3-year-old, and 7 (11.67%) from 4-year-old plants (Fig. 10b; Table S4).

These results suggest that both CYPs and UGTs exhibit the highest transcriptional activity in 5-year-old *P. japonicus*, indicating an enhanced downstream biosynthetic capacity at this stage. Overall, genes involved in all three phases of ginsenoside biosynthesis in *P. japonicus* are significantly influenced by developmental age, thereby affecting the accumulation of oleanolic-acid- and dammarane-type saponins.

### Simple sequence repeat analysis

SSR analysis using MISA software identified a total of 23 603 SSR loci from the *P. japonicus* transcriptome unigene sequences. Among these, mononucleotide repeats were the most abundant, with 7975 loci accounting for 33.79% of the total. This was followed by 7603 dinucleotide repeats (32.21%) and 5470 trinucleotide repeats (23.18%). Tetranucleotide and pentanucleotide repeats were detected at 732 and 282 loci, respectively. Hexanucleotide repeats were the least common, with only 228 loci, comprising 0.17% of all SSRs (Tables 4 and S5). These results provide a valuable foundation for future studies on germplasm identification, conservation, and molecular breeding of *P. japonicus*.

**Table 4.** Simple sequences repeat statistics of *P. japonicus*.

SSR types	Number										Total
	5	6	7	8	9	10	11	12	13	≥14	
Mononucleotide	0	0	0	0	0	5932	2043	0	0	0	7975
Dinucleotide	0	3166	2123	1407	907	0	0	0	0	0	7603
Trinucleotide	2984	1247	679	497	63	0	0	0	0	0	5470
Tetranucleotide	472	197	25	15	10	12	0	1	0	0	732
Pentanucleotide	204	24	22	12	3	4	4	8	1	0	282
Hexanucleotide	97	76	23	17	5	7		1	1	1	228
Compound	1313										1313
Total											23 603

### Transcription factor profiling

**Figure. 11.**
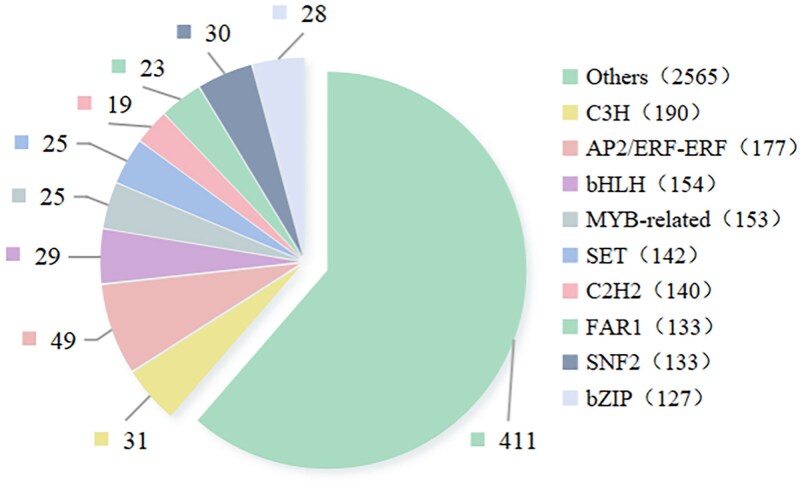
Differentially expressed TFs in the *P. japonicus* samples.

From the assembled transcription, 93 TF families, comprising a total of 3980 TF-encoding genes, were identified. Among them, the C3H family was the most abundant, with 190 genes, followed by the AP2/ERF-ERF family (177), the bHLH family (154), the MYB-related family (153), the SET family (142), and the C2H2 family (140), among others. By intersecting these TFs with 11 358 DEGs, 670 differentially expressed TFs were identified (Fig. 11). The length of the growth period may affect the accumulation of metabolites during *P. japonicus* development by modulating the expression of these TFs.

### Modules related to *Panax japonicus* saponin content

**Figure. 12.**
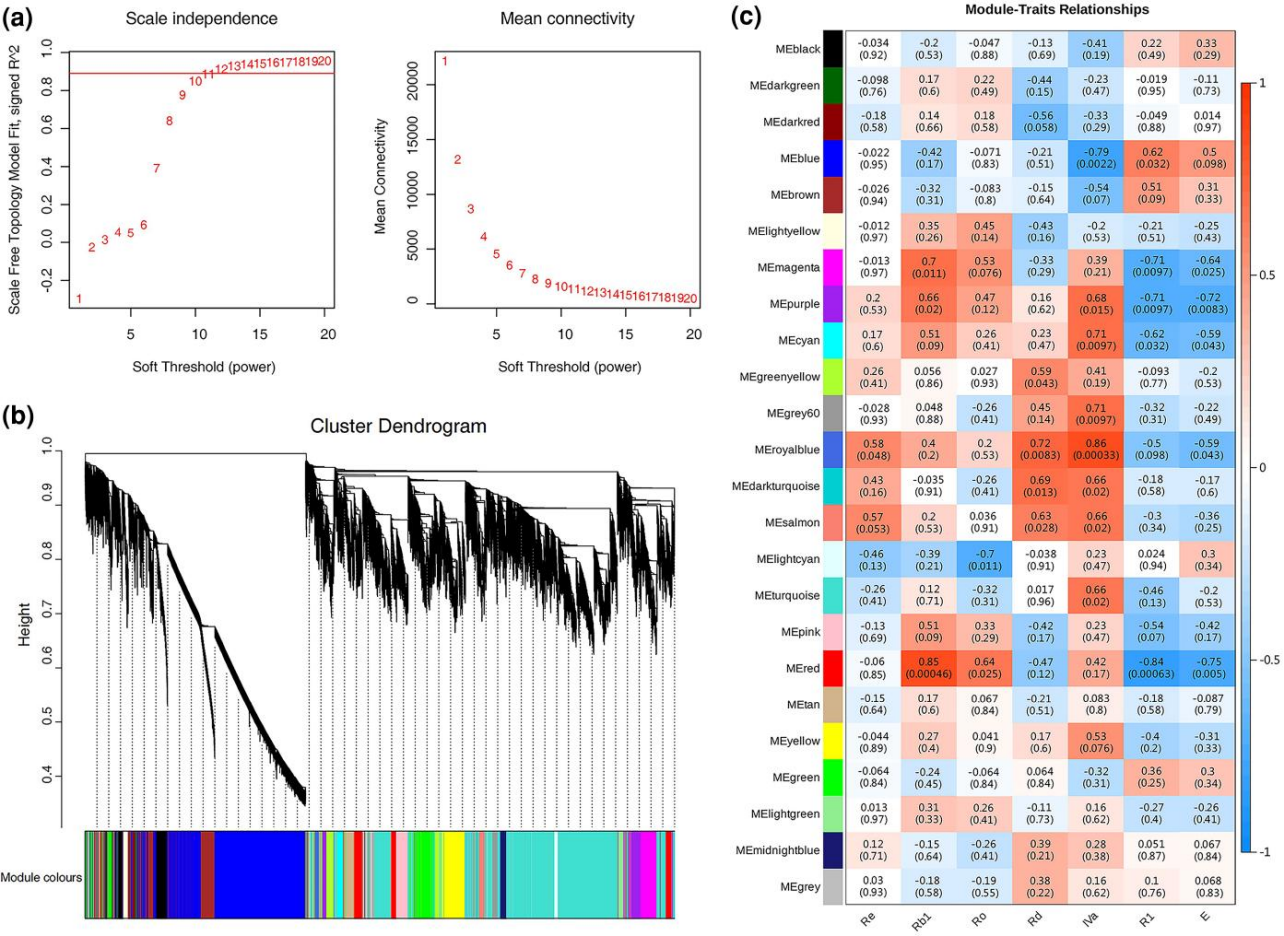
A co-expression network analysis of saponin content. (a) Network topology analysis of different soft thresholds. (b) Clustering tree based on adjacent differences and assigning module colours. (c) Building the relationship between modules and traits.

Weighted gene co-expression network analysis (WGCNA) is a systems biology method used to reveal gene association patterns across samples. We performed WGCNA alongside Pearson correlation analysis to investigate the relationship between saponin content and gene expression levels at different growth stages of *P. japonicus*. The results indicate that the adaptive index reached 0.90, and the topological model achieved an optimal balance with a relatively high mean connectivity without scale independence, while the soft-thresholding power was set to 11 (Fig. 12a). A dendrogram of gene clustering, constructed in this study, identified 22 distinct gene co-expression modules represented by different colours. The genes within the same module display analogous expression patterns (Fig. 12b). The Grey module in the general WGCNA blocks was treated as gene sets that could not be assigned to any other modules.

The results indicate that the modules MEblue, MEpurple, MEcyan, MEroyalblue, MEmagenta, MEred, and MEgrey60 exhibited strong correlations with ginsenoside content, with absolute correlation coefficients >0.7 (*P* < .05; Fig. 12c). Notably, the ‘royalblue’ module showed the highest correlation with two ginseng saponins: Ginsenoside Rd (*r* = 0.72, *P* = .0083) and Chikusetsusaponin IVa (*r* = 0.86, *P* < .001). Meanwhile, the ‘cyan’ and ‘grey60’ modules demonstrated significant correlations with Chikusetsusaponin IVa (*r* = 0.71, *P* = .0097). Furthermore, the ‘red’ module exhibited the strongest correlation with Ginsenoside Rb1 (*r* = 0.85, *P* < .001).

## Discussion

The content of active ingredients in medicinal plants is often closely linked to their growth duration (Li et al. 2020a). In this study, seven major saponin peaks were identified, including three dammarane-type saponins and four oleanolic-acid-type saponins. Among them, Ginsenoside Ro, Chikusetsusaponin IVa, Zingibroside R1, and Calenduloside E are classified as pier-type saponins, each exhibiting distinct accumulation trends over different growth years. Ginsenoside Ro represented the highest proportion of total saponin content and has been demonstrated to possess anti-cancer (Ai et al. 2024; Zhu et al. 2021) and anti-inflammatory (Xu et al. 2022) activities in clinical settings. In contrast, Calenduloside E and Zingibroside R1 were present at lower relative abundances. Notably, Calenduloside E is frequently employed in the treatment of myocardial ischaemia-reperfusion injury (Wang et al. 2022), while Zingibroside R1, derived from *Cuscuta chinensis*, has been reported to prolong the lifespan of aged *Caenorhabditis elegans* (Sayed et al. 2022). These pier-type saponins exhibit diverse pharmacological effects, which may contribute to their differential clinical applications. Furthermore, our findings reveal significant variations in saponin content among bead ginseng at different growth stages, which may, in turn, impact its therapeutic efficacy. Currently, the clinical use of bead ginseng predominantly relies on wild-harvested materials, with limited consideration given to the specific year of collection. However, studies on other traditional Chinese medicines, including *P. notoginseng* (Wei et al. 2018), *Panax vietnamensis* (Vu-Huynh et al. 2020), *Ornithogalum caudatum* (Zhao et al. 2023), Astragali Radix (Li et al. 2020b), and *Ophiopogon japonicus* (Liu et al. 2017b), have highlighted the dynamic changes in saponin composition across growth years as a key factor for quality control. Therefore, our results may serve as a valuable reference for the clinical selection and quality assessment of bead ginseng.

Comparative transcriptomic analysis revealed DEGs among *P. japonicus* plants of different growth years. Notably, the highest number of DEGs was identified in the comparison between 3- and 5-year-old plants, with 3403 genes upregulated and 3295 genes downregulated. Among the upregulated DEGs in the DY3_vs_DY5 group, members of the WRKY TF family were most prominently represented (Table S6). WRKY TFs are well-known regulators of plant defence responses (Ng et al. 2018). It is therefore hypothesized that the upregulation of WRKY genes in 5-year-old *P. japonicus* may contribute to enhanced stress adaptation during the maturation stage. In contrast, the DY3_vs_DY4 group exhibited the lowest number of DEGs, suggesting a more gradual transcriptional shift between the third and fourth growth years. These findings indicate that 5-year-old *P. japonicus* undergoes more distinct and complex transcriptional reprogramming compared with other age groups. Furthermore, KEGG pathway enrichment analysis revealed significantly enriched pathways associated with terpenoid biosynthesis, including various plant secondary metabolites, ubiquinone and other terpenoid-quinone biosynthesis, and sesquiterpenoid and triterpenoid biosynthesis. These enriched pathways may reflect age-dependent regulation of secondary metabolite production, potentially linked to the medicinal quality of *P. japonicus.*

Subsequently, we investigated the expression patterns of genes involved in the triterpenoid ‘saponin’ biosynthesis pathway in bead ginseng across different growth stages. Several genes, including *HDR1* (Cluster-83652.1), *HDR2* (Cluster-84980.1), *HDR3* (Cluster-84980.2), *IDI1* (Cluster-82476.2), and *IDI2* (Cluster-82476.3), exhibited increased expression with age, indicating their active involvement in later developmental stages. In contrast, the expression levels of *AACT2* (Cluster-79865.1), *SS1* (Cluster-95802.1), *SS2* (Cluster-95802.2), and *β-AS* (Cluster-78640.7) declined progressively with plant age. β-AS, as a rate-limiting enzyme, plays a critical role in the biosynthetic of oleanolic-acid-type saponins (Fig. 7). In a previous study, the *β-AS* gene from *P. japonicus* (Pjβ-AS) was heterologously expressed in *P. notoginseng* cells, resulting in the upregulation of key triterpenoid saponins biosynthetic genes and a significant increase in saponin content (Chen et al. 2022). Another study further demonstrated that overexpression of Pjβ-AS in *P. japonicus* cells significantly enhanced saponin biosynthesis efficiency (Zhang et al. 2019). Consistent with these findings, our results show a strong positive correlation between *β-AS* (Cluster-78640.7) expression and the accumulation of Chikusetsusaponin IVa (*R* > 0.98, *P* < .05; Tables S7). These results suggest that modulation of β-AS expression may serve as a promising strategy for enhancing saponin production through the triterpenoid biosynthetic pathway in bead ginseng. Given that Chikusetsusaponin IVa is recognized as a key quality marker in bead ginseng (Chinese Pharmacopoeia Commission 2020), our findings provide important insights for improving the quality and standardization of bead ginseng as a medicinal material.

Using WGCNA, we identified seven gene modules that exhibited a strong correlation with saponin content. A total of 375 highly expressed Tfs (FPKM > 5) from these modules are listed in Table S8. The correlation analysis between the contents of four OA-type ginsenosides (Zingibroside R1, Calenduloside E, Ginsenoside Ro, and Chikusetsusaponin IVa) and one PPT-type ginsenoside (Ginsenoside Rb1), and the expression levels of CYPs and UGTs from the MEred, Mepurple, MEblue, and MEmagentain modules are presented in the attached table. Several candidate genes with strong positive correlations (*r* > 0.8) with ginsenoside content were identified, including: *CYPs* (Cluster-64372.0, Cluster-80853.0, Cluster-90084.2) and *UGTs* (Cluster-95117.1, Cluster-93754.0, Cluster-77912.1), which were significantly correlated with Ginsenoside Rb1 content; *UGT* (Cluster-77912.1), which showed a strong correlation with Chikusetsusaponin IVa; CYPs (Cluster-90084.2, Cluster-64372.0) and *UGT* (Cluster-95117.1), which were associated with Ginsenoside Ro content (Table S9). In addition, a total of 23 603 SSR loci were identified in the transcriptome, with mononucleotide and dinucleotide repeats representing the highest proportions. These findings are consistent with the SSR distribution patterns reported by Li et al. (2020c) and Sahoo et al. (2019). The identified SSR markers may facilitate the molecular classification and genetic identification of *Panax* plants.

The global utilization of medicinal plants is rapidly expanding, primarily driven by the increasing demand for their bioactive secondary metabolites (Yang et al. 2012). Concurrently, wild populations of *P. japonicus* are undergoing a significant decline, underscoring the urgent need for sustainable strategies to preserve and utilize this valuable resource. To optimize the use of *P. japonicus* under limited cultivation conditions and time constraints, it is essential to identify approaches that enhance the accumulation of its active constituents. The data, key genes, and regulatory pathways identified in this study can provide valuable references for subsequent research on bead ginseng saponin synthesis and the elucidation of its underlying regulatory mechanisms.

## Conclusions

In this study, we quantified the ginsenoside content at different developmental stages of *P. japonicus* rhizomes and analysed the expression patterns of genes involved in saponin biosynthesis using transcriptomic profiling. Several candidate CYP450 and UGT genes potentially involved in saponin synthesis were identified, providing valuable insights into the molecular mechanisms underlying saponin biosynthesis in *P. japonicus*.

## Supplementary Material

plaf034_Supplementary_Data

## Data Availability

Transcriptome data for this study are available on the NCBI archived sequence database (SRA; http://www.ncbi.nlm.nih.gov/sra) with accession number PRJNA1177168. Supplementary data for this article is available online at *AoB PLANTS* online.
